# Rational Treatment of Smallpox

**Published:** 1899-12

**Authors:** T. C. Osborn

**Affiliations:** Cleburne, Texas


### Rational Treatment of Smallpox.

BY T. C. OSBORN, M. D., CLEBURNE, TEXAS.

Having succeeded thus far in eliciting public attention to my rational treatment of smallpox, it is perhaps proper that I should explain the theory upon which the treatment was based.

My association with the epidemic which invaded Cleburne in 1894, and witnessing the results of the treatment as laid down in the text-books, I meditated earnestly upon the pathology of the disease, and became finally convinced that it was strictly a *typical skin affection*, due to a specific germ colonizing the cells of the cuticle, requiring eighteen days for incubation—according to my record

—inducing, in the first place, a vivid exanthesis over the head and face, the neck and the chest as far down as the nipple line—no lower—which lasted until the second crop of pustules appeared. If this exanthesis is absent, and a pallor is present, it is almost conclusive evidence of some organic vice in the system, and indicates a serious prognosis in the case. On the other hand, the erythematous blush indicates a healthier condition of the system, and such cases always recover. With the exanthesis there is rarely a chill, but the temperature rises rapidly, often as high as 105° by the thermometer, lasting for forty-eight hours, before any appearance of pimpling. In the course of a few hours the pimples becomes vesicles, which in turn become pustules, with a decided areola around each matrix, giving the appearance of a mammillated tumor, the pustude arising from the center and pitted at the top. At this stage of the disease there can be no mistake in the diagnosis, as no other eruption ever has this characteristic feature. At the end of the third day of the eruption, the second crop of pustules comes out, and it is this which creates the confluent form of the disease.

In the second place, the pathogeny is to be considered. What the bacterial germ is like I do not know, but if left for me to surmise, my answer would be, the sporal germ is motile and covered with flagella, each fibril terminating in a spike sharp enough to plunge along and dip through the cuticle and rete mucosum, down to the cutis vera, where it creates the pus formation.

When the germs have passed through the cells of the cuticle into the mucous tissue, vesication is made; and when into the true skin, pustulation is established. The contagiousness of smallpox is due to actual contact in which a germ or germs pass from person to person, and act, as believed by the people, as an atmospheric infection. If, then, these two predicates be well grounded, the nature or pathology, and the cause or pathogeny—but one consequent truth can be admitted; to destroy the cause is to abort the disease.

In the third place, in considering the reliability of antiseptics, there is nothing superior to the bichloride of mercury in watery solution, when applied to regions invaded by bacteria, but owing to its toxic vehemence it is applicable only to the dermal structure. Here it may be used even to a saturated strength without danger, except in cases of idiosyncracy, and in these cases, which occur once in ten thousand instances, the only danger is from mercurial ptyalism. No case is on record where any other result is known to have happened. I admit that internally it is deadly poison. Even as a spray in the eyes, it is exceedingly distressing. And knowing this, as well as knowing that the virus of variola has a strong predilection for the eyes, frequently destroying vision, and feeling doubtful of a successful arrest of the disease with the sponge bath of bichloride alone, a happy inspiration occurred to me in the John Dodson case, to use the peroxide of hydrogen—a prompt antiseptic—in the eyes, nose, fauces, and ears to kill the germs there. The germs are very abundant in these cavities, as may be readily seen in spraying them with glycozone. Smallpox differs in its destructive features from scarlatina, in which the ears are the parts to suffer. I considered the spraying as the best part of the treatment, and subsequent practice has confirmed my belief in its essential power in managing variola, scarlatina, and varicella.

Dr. Kornitzer of Cincinnati, antedated me in suggesting the value of the bichloride of mercury *in spirit solution* in these eruptive diseases, but he gives no cases, as far as I know from his letter in the *Southwestern Medical Journal*, for September, 1896. I have never seen the pamphlet he speaks of in that letter.

My priority is backed up by reporting a case aborted by the use of bichloride of mercury in watery solution, and by peroxide of hydrogen in the facial cavities, with no outside influence from any one. If there has ever been a case recorded of such a treatment of eruptive diseases as I lay claim to, I do not know it.

But I care nothing about the priority of the treatment, and am only anxious about its adoption as the only rational management of smallpox up to the date of my existence. It is better that I should state here that quinine is the only medicine that will not disagree with the management of variola. It seems to do good, but is best given in the preliminary fever of that disease. A daily administration of hypo-sulphite of soda, thirty grains, in half a glass of water, is antiseptic in its effects, and is a pleasant saline for aperient purposes. It has seemed to me that heart failure from the persistent high temperature of the body is accountable for many cases of death from smallpox, and acting on that idea, I have commended a whiskey toddy two or more times a day. It seems very serviceable.

In a number of cases of varicella and of scarlatina which I have treated, the same rational treatment has promptly succeeded in aborting these diseases. It is my practice to protect every suspect associated with either of them, and to permit said subjects to remain in the room of an afflicted patient, fearless of any subsequent case happening in that house; and I have reason to be thankful for it. It is my earnest conviction that there need be but one case at any place, if the solution is thoroughly applied to all suspected persons, once or twice during the stage of incubation. No quarantine is necessary. Indeed the quarantine has seemed harmful, as out of the twenty-seven cases in the epidemic at Cleburne in 1894, every one of whom was vaccinated, the disease spread incontinently. Remember, my John Dodson case was among the last of that epidemic; and what I had seen inspired the idea of adopting the rational treatment.

To Dr. Cass of Cameron, I am under obligations for the interest he took in the treatment of himself, and Mr. Magee, in publishing their cases in the Cameron *Herald*, as successfully aborted. He states that there were a hundred pustules on Mr. Magee when he began the treatment, all of which—the pustules—were promptly aborted. I am also under many obligations to Dr. R. H. L. Bibb, a distinguished physician of Saltillo, Mexico, for his recent report in the Texas Medical Journal of many cases of smallpox treated by my method successfully.

At last we are assured that smallpox can be aborted.

